# Mineral licks as environmental reservoirs of chronic wasting disease prions

**DOI:** 10.1371/journal.pone.0196745

**Published:** 2018-05-02

**Authors:** Ian H. Plummer, Chad J. Johnson, Alexandra R. Chesney, Joel A. Pedersen, Michael D. Samuel

**Affiliations:** 1 Department of Forest & Wildlife Ecology, University of Wisconsin – Madison, Madison, Wisconsin, United States of America; 2 Departments of Soil Science, Chemistry, and Civil & Environmental Engineering, University of Wisconsin – Madison, Madison, Wisconsin, United States of America; 3 Molecular and Environmental Toxicology Center, University of Wisconsin – Madison, Madison, Wisconsin, United States of America; 4 Departments of Soil Science, Chemistry, and Civil & Environmental Engineering, Molecular and Environmental Toxicology Center, University of Wisconsin – Madison, Madison, Wisconsin, United States of America; Creighton University, UNITED STATES

## Abstract

Chronic wasting disease (CWD) is a fatal neurodegenerative disease of deer, elk, moose, and reindeer (cervids) caused by misfolded prion proteins. The disease has been reported across North America and recently discovered in northern Europe. Transmission of CWD in wild cervid populations can occur through environmental routes, but limited ability to detect prions in environmental samples has prevented the identification of potential transmission “hot spots”. We establish widespread CWD prion contamination of mineral licks used by free-ranging cervids in an enzootic area in Wisconsin, USA. We show mineral licks can serve as reservoirs of CWD prions and thus facilitate disease transmission. Furthermore, mineral licks attract livestock and other wildlife that also obtain mineral nutrients via soil and water consumption. Exposure to CWD prions at mineral licks provides potential for cross-species transmission to wildlife, domestic animals, and humans. Managing deer use of mineral licks warrants further consideration to help control outbreaks of CWD.

## Introduction

Chronic wasting disease (CWD) was first observed in 1967 [1] and long thought to be a disease of minor scientific curiosity affecting mule deer (*Odocoileus hemionus*) and confined to the Rocky Mountains in northern Colorado and southern Wyoming, USA. Subsequently the disease was found in white-tailed deer (*O*. *virginianus*) and elk (*Cervus canadensis*). The geographic range of CWD has also expanded dramatically since 2000 [2] and is now present in 25 U.S. states, two Canadian provinces (http://www.nwhc.usgs.gov/disease_information/chronic_wasting_disease/index.jsp), South Korea, Norway [3], and Finland (https://yle.fi/uutiset/osasto/news/first_case_in_finland_elk_dies_due_to_chronic_wasting_disease/10108115) and has been found in moose (*Alces alces*) and reindeer (*Rangifer tarandus*) [2,4]. In addition, CWD prevalence has continued to increase with some free-ranging herds exceeding 30% [5] and captive herds exceeding 80% [6]. As the prevalence of CWD increases in wild populations, declines in abundance are expected [5,7,8].

Studies in captive facilities, where deer are kept in close quarters, have demonstrated that CWD can be transmitted by direct (animal-to-animal) and indirect (environmental) routes [9]. Although environmental routes of transmission are considered an important component of CWD epizootics in free-ranging populations [10] the relative importance of direct and environmental transmission during CWD epizootics is not currently understood. In particular, the inability to measure low levels of prions in complex environmental matrices has hampered identification of potential focal sites for transmission. Prior to the present report, detection of CWD prions in naturally contaminated environments was limited to a single sample from a river in Colorado with enzootic CWD and a concurrently acquired sample in the flocculation basin of a water treatment plant [11]. Knowledge of the relative importance of direct vs. environmental transmission in free-ranging cervids is crucial for predicting the long-term dynamics of CWD outbreaks [10]. Furthermore, indirect transmission of CWD prions among cervid species through the environment seems likely and could in principle lead to transmission to additional species [12–16] with unknown consequences for humans, livestock, and wildlife.

Prions are composed of disease-associated, abnormally folded forms of the host-encoded prion protein (PrP). (In this report, we refer to the infectious prion protein associated with CWD as PrP^CWD^). Chronic wasting disease prions are distributed widely in nervous, lymphatic, blood, and muscle tissues in infected cervids and are shed via urine, feces, and saliva during an extended incubation period of 2 or more years [17–22]. Prions can also be deposited in the environment via infected carcass or viscera from infected cervids left by hunters [9,23]. Once deposited in the environment, prions can become associated with soil particles [24–26] and resist degradation for many years [9,27–29]. Furthermore, binding of prions to clay mineral particles present in soils can dramatically enhance transmission via the oral route of exposure [30–31], possibly due to increased residence time in the gastrointestinal tract and promotion of uptake by enterocytes and M cells [32]. Uptake via oronasal mucosa has recently been demonstrated as an important pathway for CWD prion entry [33], and clay particles may serve as a carrier of CWD prions via this route of exposure [34].

**Fig 1:**
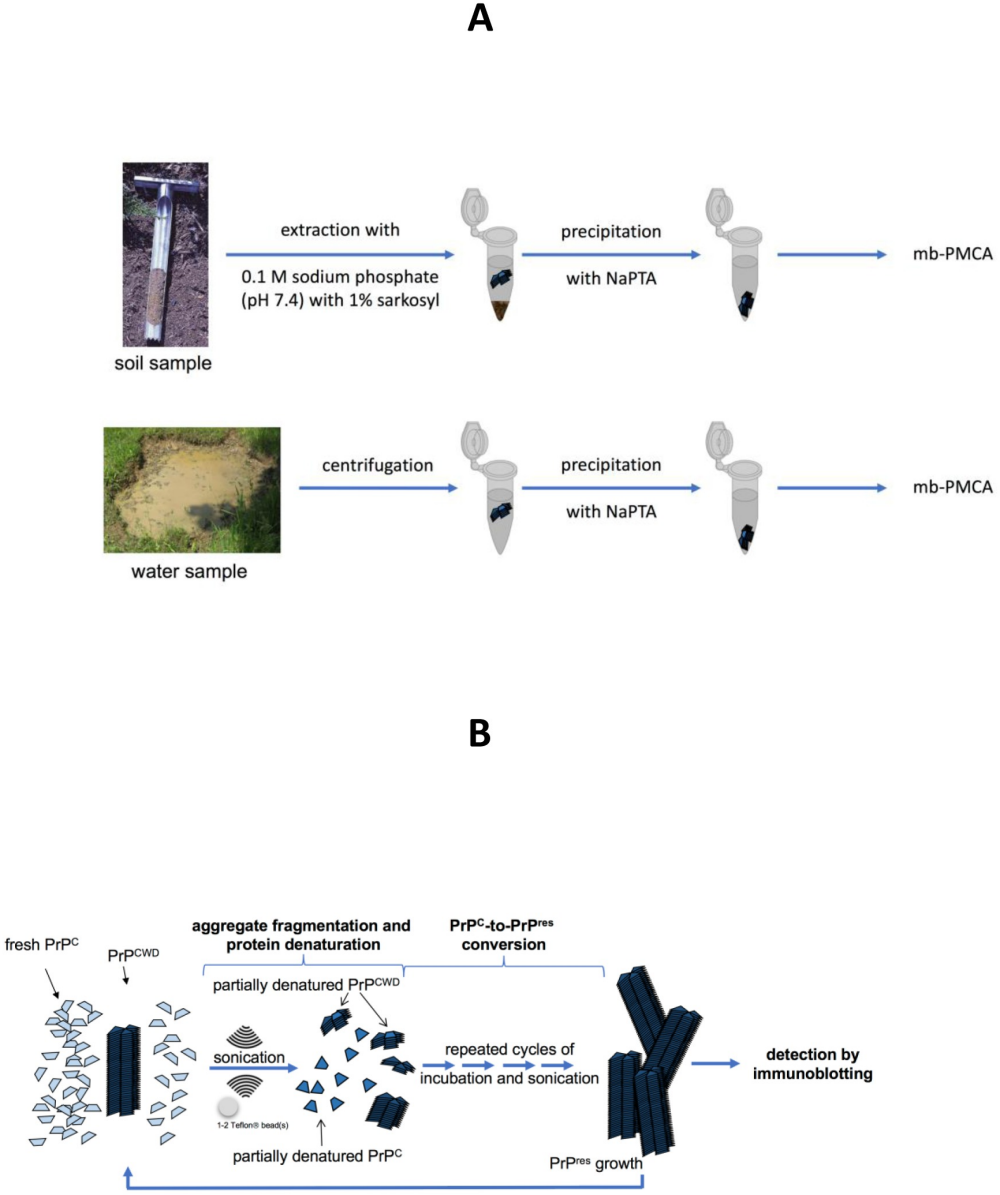
Analytical procedure to detect chronic wasting disease prions in soil and water samples. (A) Extraction of prions from soil and water samples. (B) Microplate-based protein misfolding cyclic amplification. Abbreviations: mb-PMCA, microplate-based protein misfolding cyclic amplification; NaPTA, sodium phosphotungstic acid; PrP^C^, normal, benign prion protein; PrP^CWD^, pathogenic prion protein associated with chronic wasting disease; PrP^res^, proteinase K-resistant prion protein.

Sites where cervids congregate to feed, to supplement mineral intake, or for other reasons are hypothesized to serve as foci for environmental transmission of CWD prions between infected and naïve animals [35–37]. Mineral licks contain naturally or anthropogenically enhanced concentrations of mineral nutrients that attract wildlife and livestock to satisfy their physiological nutrient requirements by consuming soil and water [38]. Deer may consume more than 0.5 kg of soil monthly from licks [39]. The use of mineral licks by deer and other species, and the continuous deposition of prions via saliva, feces and urine from infected animals [18–19,22,40–41] suggests mineral licks may serve as important focal points for environmental transmission of CWD. Currently, little is known about whether mineral licks serve as reservoirs for CWD prions or their importance in CWD transmission among cervids, other wildlife, and domestic animals. This lack of knowledge is due primarily to the absence of practical and sensitive methods to test putative environmental sources. Advances in highly sensitive *in vitro* assays (e.g., protein misfolding cyclic amplification, PMCA) have enabled detection of small amounts of CWD prions in a variety of biological matrices [42–44]. Protein misfolding cyclic amplification relies on the *in vitro* conformational conversion of the normal, benign prion protein (PrP^C^) to a proteinase K (PK)-resistant form (PrP^res^) as catalyzed by infectious prions. A sample putatively containing PrP^CWD^ is placed in an excess of PrP^C^ and is subjected to repeated cycles of sonication and incubation to amplify the amount of PrP^res^. Sensitivity is enhanced by replenishing the supply of PrP^C^ and subjecting the sample to further rounds of cyclical sonication and incubation (Fig 1).

**Fig 2:**
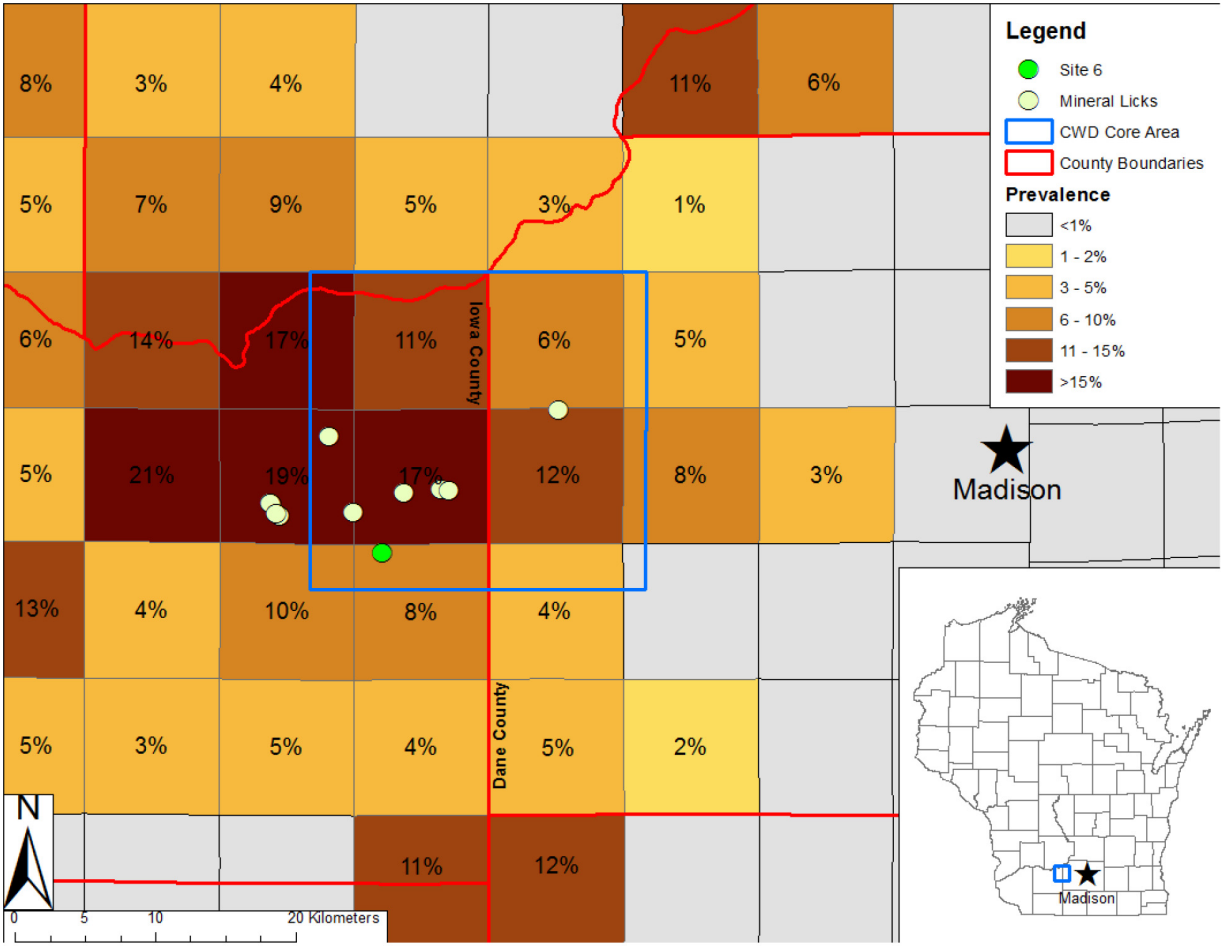
Mineral licks and chronic wasting disease prevalence. Locations of sampled mineral licks and prevalence of chronic wasting disease (CWD) in hunter-harvested white-tailed deer from 2010–2013 in south-central Wisconsin, USA. Squares are townships of 9.66 km per side. Inset shows state of Wisconsin, USA. Site 6 denotes the mineral lick with CWD-positive fecal samples.

Here, we test the hypothesis that mineral licks used by deer harbor CWD prions, thus serving as potential environmental reservoirs for these infectious agents. During 2012–2015 we collected soil and water samples from 11 mineral licks (10 man-made and one natural) frequented by free-ranging white-tailed deer in a large CWD enzootic zone west of Madison, Wisconsin, USA [6] (Fig 2). We adapted a 96-well microplate variant of PMCA that incorporates Teflon^®^ beads (mb-PMCA) [44] to detect CWD prions in soil and water samples. We optimized conditions to extract CWD prions from soils to enable reliable detection by mb-PMCA (S1 Text and S2–S4 Figs). We also tested deer feces collected in proximity to a mineral lick as a potential source of CWD prions. We previously detected CWD prions in fecal and urine samples from experimentally infected cervids by bead-assisted PMCA [22].

## Materials and methods

### Ethics statement

Field studies conducted on public lands were done with the permission of the Wisconsin Department of Natural Resources. Field studies on private lands were done with the permission of the landowners. Field studies did not involve endangered or protected species.

### Sample collection and preparation

Eleven mineral licks in the CWD-affected zone of south-central Wisconsin were located with the assistance of Wisconsin Department of Natural Resources personnel during 2013 (Fig 2). Deer visiting these mineral licks consume soil to supplement their mineral intake. During rain events, rainwater often accumulates at the lick sites. Deer visiting rainwater-filled mineral licks often stand in the water and suspend sediment as they drink. We therefore collected soil samples from dry mineral licks and, after a rainfall event, water samples before and after disturbing the underlying sediment. We collected soil at each mineral lick a single time as follows: six soil samples (2.54 cm diameter, 2.54 cm depth) were collected using a 1” diameter galvanized steel LaMotte soil sampling probe when water was not present. For each mineral lick site, the upper and lower 1.27 cm halves of the soil samples were pooled separately to yield a single pooled sample for the upper soil layer and one for the lower soil layer. Soil from Site 2 was too wet to reliably separate into upper and lower layers and was therefore pooled and analyzed as one sample. Pooled soil samples were freeze-dried, homogenized, and sieved through #18 U.S. standard testing sieve (VWR 57334–450) to remove water and non-soil components. We returned to each lick after a rainfall event and collected one water sample prior to and a second after disturbing the underlying sediment with a stick found in the immediate vicinity of each lick. Water collected prior to disturbance was clear of sediment and water collected after disturbance was turbid. We opportunistically collected fecal samples in the proximity of one heavily used lick to determine whether prions were shed in feces by deer using this site.

### Extraction of prions from environmental samples

We evaluated a series of extractant solutions for their ability to recover prions from amended soils and allow amplification by mb-PMCA (S1 Text and S1 Fig). Of the extraction solutions evaluated 0.1 M sodium phosphate (pH 7.4) with 1% (w/v) *N*-lauroylsarcosine sodium salt (sarkosyl) followed by precipitation with sodium phosphotungstic acid (NaPTA) was selected.

From each pooled upper soil sample and each pooled lower soil sample, two 25 mg soil subsamples were rinsed twice with ultrapure water (18 MΩ cm; Barnstad GenPure Pro) before extraction of soil-bound prions. Each subsample extract was used to seed four mb-PMCA replicates. The upper or lower soil sample from each mineral lick was therefore represented by four analytical replicates for each of two subsamples (8 replicates for each soil layer, 16 soil replicates per lick). Water samples (50 mL) were centrifuged (2 min, 2000*g*) to separate particulate matter before 100 μL was removed and diluted 1:1 (v/v) with 3% sarkosyl in PBS. Following, NaPTA precipitation, detailed below, each sample was used to seed four mb-PMCA replicates (4 replicates for each water sample, 8 water replicates per lick).

Prions were extracted from two independent 100 mg replicates of each deer fecal sample using a protocol for ovine feces [45] modified to include additional rounds and a longer duration of centrifuging to fully clarify samples. Each fecal sample was diluted 1:9 (w/v) in ultrapure water containing Roche complete ultra mini EDTA-free protease inhibitor (Fisher Scientific 50-100-3269) and homogenized twice for 40 s at maximum speed (6 m s^-1^) in bead-beater tubes containing glass beads and silicon carbide particles (MP Biomedical #116916100). Sodium dodecyl sulfate was added to a final concentration of 1% (v/v) before three further homogenizations by bead beating. Samples were rotated and incubated (60 min, room temperature), then centrifuged (60 min, 15000*g*, 10 °C) to clear the particulate matter. Further clarification was achieved by transferring the supernatant to a fresh tube and centrifuging under the same conditions. Supernatant was then transferred to another tube and diluted 1:1 (v/v) with phosphate-buffered saline (PBS) containing 4% sarkosyl. Pierce Universal Nuclease for Cell Lysis (Thermo Scientific #88701) was added before heating the samples to 50 °C for 30 min.

Prions in water and in extracts from soil and fecal samples were precipitated overnight with NaPTA (final NaPTA concentration of 0.57%) [46]. The following day, samples were centrifuged (30 min, 15000*g*) and the supernatant was discarded. The resultant pellets were rinsed (0.1% sarkosyl, 0.5 M EDTA) before the sample was centrifuged (10 min, 15000*g*, room temperature) and the supernatant was discarded. Pellets were resuspended in 100 μL of PMCA resuspension buffer (PBS, additional 150 mM NaCl, 4 mM EDTA, pH 8.0, 1% (v/v) Triton X-100, and protease inhibitor).

### Protein misfolding cyclic amplification

Precipitated samples were diluted 1:9 in brain homogenate from the Tg(CerPrP)1536^±^ line of transgenic cervidized mice (normal brain homogenate, NBH) expressing the dominant white-tailed deer PrP^C^ genotype in wells of a 96-well microplate containing one 2.38 mm Teflon^®^ bead per well. The sample plate was placed into a cup horn sonicator (Misonix S-4000) for 96-cycles of 20 s sonication at 46 amplitude followed by 29 min 40 s incubation at 37 °C [42–44]. After one 96-cycle round of mb-PMCA, an aliquot (4 μL) of each sample was diluted 1:9 in fresh NBH before beginning another round. Following two rounds of mb-PMCA for soil and three rounds for feces, 15 μL aliquots of each sample were digested with proteinase K (final concentration 0.2 μg∙μL^-1^) in 5.25 μL of dilution buffer (4% SDS (w/v), 0.1% (v/v) Triton X-100 in PBS) before fractionation on 15-well 10% Bis-Tris gels (Life Technologies #NP0303BOX). Separated proteins were electrotransferred to polyvinylidene fluoride membranes (Millipore #IPVH00010) and blocked for 1 h in 3% (w/v) powdered non-fat milk in Tris-buffered saline containing Tween 20 (TBST). We probed membrane bound proteins with monoclonal antibodies BAR224 (1:10000) and 8G8 (1:5000) applied in 3% milk in TBST for 1 h before rinsing the membranes with TBST. Primary antibodies were detected with horseradish peroxidase conjugated goat anti-mouse IgG (1:20000 in 3% milk in TBST) and Supersignal West Pico chemiluminescent substrate (Thermo Scientific #34080).

All mb-PMCA experiments included eight replicates of unspiked NBH as a negative control for spontaneous formation of PrP^res^ during mb-PMCA, a total of 42 replicates in 5.5 plates (2 replicates had incomplete PK digestion and, thus, were inconclusive). Because no extraction was performed on the water, unspiked NBH also served as a negative control for the water. In addition, to control for the possible effect of soil extracts on promoting formation of PrP^res^, we extracted Elliot silt loam (International Humic Substances Society, St. Paul, Minnesota, United States) and treated the extract in the same manner as the extracts from the mineral licks; two concurrent independent extracts were used to seed 4 replicates apiece and put through 2 rounds of mb-PMCA. Feces from a white-tailed deer negative for CWD were put through the fecal extract protocol and two rounds of mb-PMCA to serve as a negative control and validate the fecal extraction methods; two concurrent independent extracts were used to seed 4 replicates apiece and put through 2 rounds of mb-PMCA. For positive controls, we included the 1:3.1×10^4^ and 1:1.2×10^17^ dilutions of a 10% (w/v in PBS) brain homogenate of an orally inoculated, clinically affected CWD-positive white-tailed deer (96 GG) on every plate. We reliably detected prions in the 1:1.6×10^5^ dilution of the CWD-positive brain homogenate after one round of mb-PMCA and in the 1:7.5×10^19^ dilution after two rounds.

### Contamination controls

We took several precautions to prevent inadvertent introduction of CWD prions into the field samples during sample collection and continuing through mb-PMCA testing. These precautions included changing gloves between collection of fecal samples; changing gloves between mineral licks; wiping the LaMotte soil sampling tool with 10% bleach before and after each mineral lick; discarding plastic bags used for soil and fecal collection after a single use; discarding 50 mL water collection tubes after a single use; double bagging of samples at the site; aliquoting stock solutions to single-use volumes; changing pipette tips after each use; wiping lab tools with 10% bleach before and after each use; using fresh disposable paper bench covering for every task; handling and loading plates with only one sample at a time and changing gloves between samples; loading plates with all wells capped except those being seeded with that replicate; and handling samples and loading plates in the following order: no seed negative controls, matrix extract negative controls, experimental samples, followed by positive controls.

## Results

We detected CWD prions in soil samples, water samples, or both collected from nine of the 11 mineral licks following two rounds of amplification with mb-PMCA (Table 1). We limited the number of mb-PMCA rounds to two for soil and three for feces to minimize the possibility of *de novo* prion generation [47]. Soil from seven licks contained CWD prions. No pattern was apparent in the presence of prions in the upper vs. lower 1.27 cm of soil. We sampled water from nine mineral licks. Prions were detectable in the undisturbed water from four of the sites and in disturbed water from two of these sites. Two mineral licks (Sites 2 and 6) contained detectable CWD prions in both water and soil samples. The amounts of prions amplified from these soil and water samples was near the limit of detection for two rounds of mb-PCMA, likely due in part to co-extracted inhibitors of the PMCA reaction and incomplete extraction from soil particles. The detection of prions at 9 of 11 sites sampled, however, demonstrates widespread contamination of mineral licks in the CWD outbreak zone. The generally higher detection frequency of CWD prions in water samples relative to the corresponding soil samples suggests that either prion concentrations are higher in the water samples or that co-extracted constituents from soil inhibited amplification by mb-PMCA.

**Table 1:** Detection of CWD prions at mineral lick sites in the CWD-affected area of south-central Wisconsin, USA (see Fig 1)^†^.

site	detection	soil layer^‡^		water^‡^	
		replicates positive for CWD prions^‡^			
		upper	lower	undisturbed	disturbed
1	+	1	0	0	0
2	+	NA	1^§^	1	0
3	–	0	0	0	0
4	–	0	0	0	0
5	+	0	0	2	1
6	+	0	1	3	1
7	+	0	0	1	0
8	+	0	2	0	NA^¶^
9	+	1	0	NA^¶^	NA^¶^
10	+	0	1	NA^¶^	NA^¶^
11	+	1	0	0	0
**total sites positive**	**9**	**3**	**4**	**4**	**2**

^†^ Soil and water samples from each mineral lick were collected on a single occasion, respectively, and divided into subsamples and replicates for mb-PMCA analyses.

^‡^ Number of CWD prion detections from 8 (soil) or 4 (water) replicate samples of each type; upper and lower soil refer to top and bottom 1.27 cm of 2.54 cm deep soil samples; NA, sample type unavailable.

^§^ Soil from site 2 was too wet to reliably split into upper and lower halves and was therefore tested as a whole.

^¶^ Only undisturbed water was tested from site 8 because not enough water was present to gather both sample types; no water was available from sites 9 and 10.

At the mineral lick site with the highest detection of CWD prions in environmental samples (Site 6), we opportunistically sampled white-tailed deer fecal pellets. We detected CWD prions in six of the 10 fecal samples after three rounds of amplification by mb-PMCA. Of eight replicates tested for each fecal sample, one fecal sample had four positive replicates, three had two positive replicates, and two had a single positive replicate.

Importantly, no false positives were produced in any of our negative control samples. No PrP^res^ was detected after two or three rounds of amplification by mb-PMCA in negative control samples conducted when testing lick samples, which included at least one no-seed control, one negative soil extract control, and one no-seed fecal extract control from a CWD-negative white-tailed deer.

## Discussion

Our results demonstrate that CWD-infected white-tailed deer deposit prions at mineral licks they visit. Although the mechanism of prion deposition is unknown, we suspect deposition of saliva by infected deer during ingestion of soil and water at mineral licks has the highest potential to facilitate indirect transmission to susceptible deer. Saliva from white-tailed deer infected with CWD contains on the order of 1–5 infectious doses (ID_50_) per 10 mL as quantified by real-time quaking-induced conversion, where an ID_50_ is the dose of CWD prions capable of infecting half of the transgenic mice expressing cervid prion protein [48]. Frequent visitation by infected cervids could allow mineral licks to become potential “hot spots” for indirect transmission of CWD [49]. Currently, little is known about the relative importance of direct contact and environmental routes of CWD transmission in free-ranging cervids [10]. Thus, how artificial and natural mineral licks contribute to current and future CWD infection in cervids and whether licks should be managed to control cervid use are important questions for further research.

Despite the relatively recent detection of CWD in Wisconsin (2001) and the moderate incidence of infection (6–19% prevalence in adult deer in the area sampled at the time of sample collection), our results suggest contamination of mineral licks in the CWD outbreak zone is widespread. This finding suggests that mineral licks may serve as reservoirs of CWD prions that contribute to disease transmission to susceptible animals. Although the levels of CWD prions in the samples analyzed appears low, we note that the association of prions with clay minerals often present at mineral licks can dramatically enhance disease transmission via the oral route of exposure [30–31]. For hamster-adapted scrapie prions binding to montmorillonite clay particles enhanced transmission by a factor of 680, however, an upper bound on the enhancement factor could not be assigned [30–31]. At present, the degree to which binding to clay mineral particles enhances CWD transmission to deer via the oral (or nasal) route of exposure is not known. Furthermore, repeated oral exposure to prions is associated with increased likelihood of disease transmission [50]. Differences in the sialyation status of N-linked glycans between brain-derived and secreted/excreted PrP^CWD^ may impact oral infectivity [51]. Cervid species that avoid interspecific contact make use of the same mineral lick sites [49], potentially leading to interspecies transmission. Mineral licks also attract livestock and other wildlife that supplement mineral intake via soil and water consumption, exposing these animals to CWD prions. Exposure of predators and scavengers to CWD prions via consumption of infected tissue has been previously documented [23]; our results suggest that environmental exposure of non-cervid animal groups can also occur via environmental routes.

We also detected CWD prions in fecal samples collected in proximity to a mineral lick, indicating that fecal excretion represents a route of CWD deposition into the environment with potential transmission to susceptible cervids [19]. Deposition of fecal pellets by white-tailed deer near bait sites increases with higher deer visitation [52] and similar patterns probably occur at mineral licks. Thus, increased local fecal deposition by CWD-infected deer likely contributes to increased environmental concentrations of prions in and around mineral licks. Deer generally avoid consumption of feces [52]; however, the apparent long-term duration of prion infectivity in the environment [27–29], the enhanced disease transmission by soil-bound prions combined with the repeated visitation, long-term existence of and multi-generational use of mineral licks suggest the impact of concentrated environmental contamination on the dynamics of disease transmission warrants further investigation. Recent laboratory research indicates plants grown in prion-contaminated soil can accumulate prions [53]. Our data suggest that plants growing near contaminated mineral licks may warrant investigation as a source of prions for foraging animals. Areas where cervids congregate for mineral consumption, feeding and baiting sites, winter yarding, wallows [54] or other activities where CWD prions are deposited in the environment may also provide potential long-term reservoirs for transmission to cervid and non-cervid species.

## Conclusions

We used mb-PMCA to detect CWD in soil and water from mineral licks naturally contaminated with prions and used by free-ranging deer, livestock, and non-cervid wildlife species. Detection of prions in environmental reservoirs represents an important first step in understanding the contribution of environmental transmission to CWD epizootics and potential for cross-species transmission. The present study characterized an environmental prion reservoir by (1) identifying an apparent “hot spot” of deposition and potential exposure to both cervid and non-cervid species; (2) indicating CWD prions shed by free-ranging cervids are present in areas of frequent use leading to environmental contamination and potentially plant uptake; and (3) motivating investigation of the exposure and susceptibility of non-cervid species to CWD contaminated soil, water, and plant materials. Future research should be directed at quantifying CWD prion concentrations at mineral licks and other areas where cervids congregate, determining the persistence of prion infectivity at these sites, delineating spatial-temporal patterns of environmental prion deposition and accumulation, and assessing consumption by susceptible animals. Identifying additional environmental reservoirs of CWD prions and determining the contributions of direct and indirect transmission over the course of CWD outbreaks represent key aims in advancing understanding of long-term CWD infection dynamics.

## Supporting information

S1 Text Supplementary methods. (DOCX)

S1 Fig Extraction of natural organic matter (NOM) from soils and interference with immunoblot detection of PrP^CWD^. (A) Estimated amount of NOM extracted from Defore and Elliot soils using the indicated buffers. Samples of Defore or Elliot soil (25 mg) were rinsed with ultrapure water (200 μL each) with shaking (2 h, 1,200 RPM, room temperature). Samples were centrifuged (1000*g*, 10 min), and the supernatant was removed and saved as water rinse. The indicated extraction solution was added (200 μL) to each of the soil pellets and vortexed (2 h, 1,200 RPM, room temperature). Soil particles were sedimented (1000*g*, 10 min), and the supernatants were retained. Extractions were done in triplicate, and 100 μL of each extract and the water rinses were used to assay the absorbance at λ = 465. Absorbances were compared to dilution series of Elliot soil humic acid in each of the above extraction solutions, and the concentration of NOM was estimated. Shown are the mean NOM concentrations for the three replicates with the standard deviations. (B) Extraction of PrP^CWD^ from soil. PK-treated 10% brain homogenate from a CWD-positive white-tailed deer (40 μL) was adsorbed to Elliot soil (25 mg) in ultrapure water (100 μL, 24 h) followed by a 2-h desorption step in 100 μL water (to remove any non-adsorbed unbound PrP^CWD^). The sorbed PrP^CWD^ was extracted at room temperature with 200 μL of the indicated extraction solution and analyzed by immunoblotting. Abbreviations: A-G, extraction solutions (see descriptions above); M, molecular mass marker; mAb, monoclonal antibody; rinse, water rinse; S, supernatant from binding experiment.
(TIF)

S2 Fig Influence of extraction solution and soil extracts on PMCAb. (A) Extraction solutions (8 μL) were mixed with 2 μL from the second 5-fold dilution of 10% brain homogenate (BH) from an end-staged CWD-positive wt/wt deer. Normal brain homogenate (NBH; 90 μL) was added with two Teflon^®^ beads in a 200-μL thin-walled PCR tube. Samples were sonicated for 96 cycles (30 s sonication with 27:30 incubation at 37 °C between sonications). (B) Elliot soil or Defore soil (25 mg) was extracted using 100 μL of the indicated extraction solution. An aliquot (8 μL) of these extracts were mixed with 2 μL from the second 5-fold dilution of 10% BH from an end-stage CWD-positive wt/wt deer. NBH (90 μL) was added with two Teflon^®^ beads in a 200-μL thin-walled PCR tube. Samples were sonicated for 96 cycles (30 s sonication with 27:30 incubation at 37 °C between sonications). Extraction solutions are described in the text. Proteinase K (PK) resistant prion protein was detected using Western blot with antibodies 8G8 and BAR224.
(TIF)

S3 Fig Effect of humic and fulvic acid detection of PrP^CWD^ by PMCAb. Prions (10% CWD brain homogenate) were diluted using four serial fivefold dilutions in normal brain homogenate (NBH) to obtain the seed dilution used in this experiment. The seed dilution (2 μL) was transferred into 36 μL NBH with saponin, and 2 μL humic or fulvic acid (dissolved in ultrapure water) was added to achieve a total mass of 5, 2.5, 1.5, 1.25, 1, 0.75, 0.5, 0.25, 0.15, 0.1, 0.05 or 0 μg of the indicated humic or fulvic acid. All samples were subjected to a single round of mb-PMCA for 96 cycles. Immunoblots shown are representative of three replicates and were probed with antibodies BAR224 and 8G8.
(TIF)

S4 Fig Sodium phosphotungstic acid (NaPTA) precipitation reduces the inhibitory effect of soil extracts on PMCAb. After NaPTA precipitation, 10 μL sample extracts from each extraction solution indicated were added to a PMCAb reaction. Extraction solutions were (A) 0.1 M sodium phosphate buffer, pH 7.4; (C) 1% NP40 in 0.1 M sodium phosphate buffer, pH 7.4; (D) McDougall’s buffer, pH 8.23; (E) PMCA buffer (1% Triton X-100 in PBS, pH 7.4 with 0.15 M NaCl and 0.05% saponin); and (F) 1% sarkosyl in 0.1 M sodium phosphate buffer, pH 7.4. Extraction solutions (B) and (G) not in immunoblot. Samples, normal brain homogenate (NBH; 90 μL), and two Teflon^®^ beads were added to a 200-μL thin-walled PCR tubes containing NBH. Samples were sonicated for 96 cycles (0.5 min sonication with 27.5 min incubation at 37 °C between sonications). Proteinase K (PK) resistant prion protein was detected using immunoblotting with antibodies 8G8 and BAR224.
(TIF)
