# Supplementary material for: Mineral licks as environmental reservoirs of chronic wasting disease prions
Source: PLoS One. 2018 May 2;13(5):e0196745. doi: 10.1371/journal.pone.0196745 (PMC5931637; doi:10.1371/journal.pone.0196745)
Supplement: S1 Text — (DOCX) [file pone.0196745.s001.docx]

**Supplementary Methods**

We evaluated a series of extractant solutions for their ability to recover prions from amended soils and allow amplification by protein misfolding cyclic amplification (PMCA). Natural organic matter occurs at variable concentrations in soils and may be co-extracted with PrP^CWD^. We previously demonstrated that natural organic matter (NOM) in soil impacts PrP^CWD^ detection by immunoblotting [1]. Methods commonly used to extract pathogenic prion protein from soils release substantial amounts of NOM, and NOM inhibits immunoblot signal for prion protein [1-2]. The degree of immunoblot interference for both deer and hamster prions increased with increasing NOM concentration and decreasing NOM polarity [1]. These findings raise the concern that NOM co-extracted with PrP^CWD^ might interfere with detection by PMCA. We examined the degree to which NOM interferes with PMCA and the amount of NOM extracted by several PrP^CWD^ potential extraction solutions.

**Transgenic mice**

All animals were cared for in accordance with protocols approved by the Institutional Animal Care and Use Committee of the University of Wisconsin – Madison (Assurance Number A3464-01). Brain tissue from uninfected transgenic mice hemizygous for the cervid prion gene (Tg(CerPrP)1535^±^ mice) [3] was used to prepare normal brain homogenate (NBH) to use as the source for the PrP^C^ in PMCA reactions. Mice were euthanized by CO_2_ asphyxiation and immediately perfused with 1× modified DPBS without Ca^2+^ or Mg^2+^ (Thermo Scientific, amended with 5 mM EDTA). Brains were rapidly removed, flash frozen in liquid nitrogen, and stored at −80 °C until use. Brain tissue was homogenized on ice to 10% (w/v) in PMCA conversion buffer (Ca^2+^- and Mg^2+^-free DPBS, pH 7.4, supplemented with 150 mM NaCl, 1% Triton X-100, 0.05% saponin (Mallinckrodt), 5 mM EDTA, and 1 tablet Roche Complete EDTA-free protease inhibitors cocktail (Fisher) per 50 mL conversion buffer). Brain homogenates were clarified by centrifugation (2.75 min, 2,000*g*, 4 °C). Supernatant was transferred to pre-chilled microcentrifuge tubes, flash frozen in liquid nitrogen, and stored at −80 °C until use.

**Preparation of prion source**

Tissue was isolated from the brain of an experimentally inoculated white-tailed deer homozygous in glycine at codon 96 (wt/wt) showing clinical signs of CWD prior to being euthanized [4]. Brain tissue was homogenized to a concentration of 10% w/v in 1× Dulbecco’s phosphate buffered saline (DPBS) without Ca^2+^ or Mg^2+^ (137 mM NaCl, 8.1 mM HNa_2_PO_4_, 1.47 mM H_2_PO_4_; pH 7) and stored at −80 °C until use. Prior to addition to soils, CWD-positive brain homogenate (BH; 50 µL) was treated with 50 µg∙mL^-1^ (final concentration) proteinase K (PK; 60 min, 37 °C) to eliminate PrP^C^ and to *N*-terminally truncate PrP^CWD^. Proteinase K activity was halted by addition of phenylmethanesulfonyl fluoride to a final concentration of 4 mM.

**Soil samples**

Two soils contrasting in their content of natural organic matter (NOM) were used to optimize prion recovery for detection by PMCA: Elliot silt loam soil (fraction of organic carbon, *f*_oc_ = 0.029, International Humic Substances Society) and Defore silt loam soil (*f*_oc_ = 0.133, Outagamie County, Wisconsin). Concentrations of natural organic matter in soil extracts were estimated as previously described [1]. Briefly, absorbance spectra (250-700 nm) were acquired by a UV-3600 Shimadzu spectrophotometer. Triplicate samples were quantified against a five-point calibration curve (*R*^2^ > 0.98) produced with 0.005 to 1 g L^-1^ Elliot soil humic acid (1S102H; International Humic Substances Society). Samples with absorbance outside the linear range of the standard curve were diluted and reanalyzed.

**Extraction experiments**

Proteinase K-treated CWD-positive brain homogenate was allowed to interact with soils (25-50 mg) at room temperature in the presence of 100 µL ultrapure water (18 MΩ∙cm; Barnstead GenPure Pro) for 24 h followed by a 2 h desorption step in 100 µL water (to remove any non-adsorbed prion protein). The soil samples were then extracted at room temperature with 200 µL of one of the following extraction solutions: (A) 0.1 M sodium phosphate buffer, pH 7.4; (B) 0.1 M sodium phosphate buffer pH 8; (C) 1% nonyl phenoxypolyethoxylethanol (NP40) in 0.1 M sodium phosphate buffer, pH 7.4; (D) McDougall’s buffer, pH 8.23; (E) PMCA buffer (1% Triton X-100 in PBS, pH 7.4 with additional 0.15 M NaCl and 0.05% saponin) (24); (F) 1% sarkosyl in 0.1 M sodium phosphate buffer, pH 7.4; or (G) 1% sarkosyl in 0.1 M sodium phosphate buffer, pH 8. Eluates were precipitated with sodium phosphotungstic acid (NaPTA). Sodium phosphotungstic acid (4%, no Mg^2+^) was added to sample extracts to a final concentration of 0.30% [], and the extracts were incubated at 37 °C overnight. After precipitation, samples were centrifuged (30 min, 16000*g*, room temperature). The pellet was washed once with 200 µL DPBS, 0.1% sarkosyl and 50 µL 250 mM EDTA and sedimented by centrifugation (10 min, 16000*g*, room temperature). Pellets were resuspended in PMCA resuspension buffer (PBS, additional 150 mM NaCl, 4 mM EDTA, pH 8.0, 1% (v/v) Triton X-100 and miniprotease inhibitor) to 100 µL. Extracted PrP^CWD^ was analyzed by immunoblotting or bead-assisted protein misfolding cyclic amplification (see below) [6].

**Immunoblot detection of prion protein**

Prions were detected via SDS-PAGE as previously described [1]. Briefly, a 20 μL aliquot was mixed with 10 μL 10× SDS sample buffer (100 mM Tris, pH 8, 10% SDS, 7.5 mM EDTA, 100 mM dithiothreitol, 30% glycerol), vortexed, and heated (10 min, 100 °C) and fractionated on a 12% bis-tris polyacrylamide gels (BioRad). Proteins were electrotransferred from the gel to a 0.45 µm polyvinyl difluoride membrane (Millipore), blocked with 5% nonfat dry milk prepared in 1× Tris-buffered saline containing 0.1% Tween 20 for 60 min and probed with primary antibodies overnight at 4 °C. We used a combination of monoclonal antibodies (mAbs) 8G8 (Cayman Chemical, 1:1000, epitope: 97-102) and BAR224 (Cayman Chemical, 1:10000, epitope: 141-151) to probe the membrane-bound cervid PrP. Detection was achieved by horseradish peroxidase-conjugated goat-anti-mouse immunoglobulin G (BioRad, 1:10,000 dilution) using Super Signal West Pico chemiluminescent substrate (Pierce Biotechnology) to visualize protein bands. Densitometric analysis of immunoblot bands was conducted using Image J Software.

**Protein misfolding cyclic amplification**

Protein misfolding cyclic amplification was conducted as previously described [6-8]. The two versions of PMCA used, bead-assisted PMCA (PMCAb) and microplate-based PMCAb (mb-PMCA), differ in the quantity of seed and NBH substrate, number of Teflon beads per reaction, and vessel used. The seeds for initiating PMCA reactions were 10 µL (PMCAb) or 4 µL (mb-PMCA) aliquots from CWD samples in indicated experiments which were added to 90 µL (PMCAb) or 36 µL (mb-PMCA) NBH in 0.2 mL thin-walled PCR tubes (PMCAb) or 96-well PCR microplate (mb-PMCA; Axygen, Union City, CA, USA) with two (PMCAb) or one (mb-PMCA) 2.38 mm Teflon^®^ bead (McMaster-Carr, #9660K12). A dilution series was prepared by serially diluting 10% CWD BH (made up in DPBS) five-fold in NBH. Sample dilutions were used to seed reactions in the same experiment and acted as positive controls. Where indicated, CWD agent was added to normal brain homogenate from uninfected brain homogenate from Tg(CerPrP)1535^±^ mice and used as an NBH carrier to verify the success of PTA precipitation. Negative controls were NBH. Experimental plates were placed in a rack in a Misonix S-4000 microplate horn, and the reservoir was filled with ultrapure water. Each round of PMCAb or mb-PMCA consisted of 96 cycles (30 s sonication at 40-60% of maximum power, 1770 s incubation at 37 °C). At the completion of 96 cycles, new NBH was reseeded with 10 µL (PMCAb) or 4 µL (mb-PMCA) of the reaction product for serial PMCA. After completing the last round of PMCA, 20 μL of each sample was digested with PK (50 μg∙mL^-1^, 1 h, 37 °C), and the resulting PrP^res^ was analyzed by SDS-PAGE with immunoblot detection (*vide supra*).

**Supplementary results**

**Extraction solutions and extraction of natural organic matter from soil**

More NOM was extracted from the Defore than the Elliot soil for all extraction solutions (Fig S1A), consistent with the higher organic carbon content of the former. The concentration of NOM extracted from the Elliot soil was 16 to 117 µg∙mL^-1^; that from the Defore soil ranged from 56 to 1150 µg∙mL^-1^. For both soils, McDougall’s buffer (pH 8.23; extraction solution D) and PMCA buffer (pH 7.4; extraction solution E) removed the smallest amount of NOM from the soils (Fig S1A). Extraction solutions containing sarkosyl (1%; extraction solutions F and G) produced the highest immunoreactivity when PrP^CWD^ was recovered from Elliot soil (Fig S1B) despite containing the largest amount of NOM (Fig S1A).

**S1 Fig. Extraction of natural organic matter (NOM) from soils and interference with immunoblot detection of PrP^CWD^.** (A) Estimated amount of NOM extracted from Defore and Elliot soils using the indicated buffers. Samples of Defore or Elliot soil (25 mg) were rinsed with ultrapure water (200 µL each) with shaking (2 h, 1,200 RPM, room temperature). Samples were centrifuged (1000*g*, 10 min), and the supernatant was removed and saved as water rinse. The indicated extraction solution was added (200 µL) to each of the soil pellets and vortexed (2 h, 1,200 RPM, room temperature). Soil particles were sedimented (1000*g*, 10 min), and the supernatants were retained. Extractions were done in triplicate, and 100 µL of each extract and the water rinses were used to assay the absorbance at λ = 465. Absorbances were compared to dilution series of Elliot soil humic acid in each of the above extraction solutions, and the concentration of NOM was estimated. Shown are the mean NOM concentrations for the three replicates with the standard deviations. (B) Extraction of PrP^CWD^ from soil. PK-treated 10% brain homogenate from a CWD-positive white-tailed deer (40 µL) was adsorbed to Elliot soil (25 mg) in ultrapure water (100 µL, 24 h) followed by a 2-h desorption step in 100 µL water (to remove any non-adsorbed unbound PrP^CWD^). The sorbed PrP^CWD^ was extracted at room temperature with 200 µL of the indicated extraction solution and analyzed by immunoblotting. Abbreviations: A-G, extraction solutions (see descriptions above); M, molecular mass marker; mAb, monoclonal antibody; rinse, water rinse; S, supernatant from binding experiment.

**Optimization of PMCA for detection of CWD prions from soils**

To investigate the degree to which soil constituents extracted by the extraction solutions interfered with the PMCA reaction, we extracted the Elliot and Defore soils with each solution and added extracts to PMCA reactions (Fig S2A). Solution E extracts contained among the lowest amounts of NOM, and as expected, allowed the highest degree of prion amplification from Elliot soil. Of extraction conditions tested, those that included 1% sarkosyl (extraction solutions F and G) appeared to result in the largest recovery of PrP^CWD^ as determined by immunoblotting (Fig S1B) but interfered with PrP^CWD^ detection by PMCAb (Fig S2A).

**S2 Fig. Influence of extraction solution and soil extracts on PMCAb.** (A) Extraction solutions (8 µL) were mixed with 2 µL from the second 5-fold dilution of 10% brain homogenate (BH) from an end-staged CWD-positive wt/wt deer. Normal brain homogenate (NBH; 90 µL) was added with two Teflon® beads in a 200-µL thin-walled PCR tube. Samples were sonicated for 96 cycles (30 s sonication with 27:30 incubation at 37 °C between sonications). (B) Elliot soil or Defore soil (25 mg) was extracted using 100 µL of the indicated extraction solution. An aliquot (8 µL) of these extracts were mixed with 2 µL from the second 5-fold dilution of 10% BH from an end-stage CWD-positive wt/wt deer. NBH (90 µL) was added with two Teflon® beads in a 200-µL thin-walled PCR tube. Samples were sonicated for 96 cycles (30 s sonication with 27:30 incubation at 37 °C between sonications). Extraction solutions are described in the text. Proteinase K (PK) resistant prion protein was detected using Western blot with antibodies 8G8 and BAR224.

We next tested the effect of soil extracts on detection of PrP^CWD^ by PMCAb. Detection of PrP^CWD^ by PMCAb was decreased when soil extracts were included in the reaction (Fig S2B) compared to the extract solutions alone (Fig S2A) indicated an inhibitor of PMCAb eluted from the soil. The inhibition was stronger for extracts of the Defore soil. The Defore soil extracts contained a much higher level of NOM than did those of the Elliot soil (Fig S1A). We hypothesized that NOM contributed to the inhibition of PMCAb.

We therefore investigated the effect of major, operationally defined fractions of NOM (viz. humic and fulvic acids) on amplification of PrP^CWD^ from diseased brain tissue. We used Elliot soil humic acid, Suwanee River humic and fulvic acid, Leonardite humic acid, and Pahokee peat humic acid, well-characterized, reference humic and fulvic acids that are frequently used in environmental chemistry studies. We determined that humic and fulvic acids inhibited the first round of PMCAb in a concentration-dependent manner with the largest inhibition seen in samples with >0.25 μg humic acid (i.e., >6.25 µg∙mL^-1^) and >1 μg fulvic acid (i.e., >25 µg∙mL^-1^) (Fig S3). These results are consistent with NOM constituents contributing to the inhibition of PMCAb noted above.

**S3 Fig. Effect of humic and fulvic acid detection of PrP^CWD^ by PMCAb.** Prions (10% CWD brain homogenate) were diluted using four serial fivefold dilutions in normal brain homogenate (NBH) to obtain the seed dilution used in this experiment. The seed dilution (2 µL) was transferred into 36 µL NBH with saponin, and 2 µL humic or fulvic acid (dissolved in ultrapure water) was added to achieve a total mass of 5, 2.5, 1.5, 1.25, 1, 0.75, 0.5, 0.25, 0.15, 0.1, 0.05 or 0 µg of the indicated humic or fulvic acid. All samples were subjected to a single round of mb-PMCA for 96 cycles. Immunoblots shown are representative of three replicates and were probed with antibodies BAR224 and 8G8.

We therefore examined the ability of NaPTA precipitation to reduce the inhibition of these extracts. When coupled with NaPTA precipitation, one round of PMCA allowed detection of PrP^CWD^ with all solutions tested regardless of whether NBH was used as a carrier to aid in precipitation by NaPTA (Fig S4). Subsequent analyses of soil samples collected from the field were extracted with 1% sarkosyl in 0.1 M phosphate buffer, pH 7.4 (extraction solution F) followed by a NaPTA precipitation step to isolate the extracted PrP^CWD^. This extraction solution was selected due the high recovery of PrP^CWD^ from soil (Fig S1B) and the ability to eliminate interference with the PMCA reaction via NaPTA precipitation. This extract solution was previously used by Cooke et al. [9].

**S4 Fig. Sodium phosphotungstic acid (NaPTA) precipitation reduces the inhibitory effect of soil extracts on PMCAb.** After NaPTA precipitation, 10 µL sample extracts from each extraction solution indicated were added to a PMCAb reaction. Extraction solutions were (A) 0.1 M sodium phosphate buffer, pH 7.4; (C) 1% NP40 in 0.1 M sodium phosphate buffer, pH 7.4; (D) McDougall’s buffer, pH 8.23; (E) PMCA buffer (1% Triton X-100 in PBS, pH 7.4 with 0.15 M NaCl and 0.05% saponin); and (F) 1% sarkosyl in 0.1 M sodium phosphate buffer, pH 7.4. Extraction solutions (B) and (G) not in immunoblot. Samples, normal brain homogenate (NBH; 90 µL), and two Teflon® beads were added to a 200-µL thin-walled PCR tubes containing NBH. Samples were sonicated for 96 cycles (0.5 min sonication with 27.5 min incubation at 37 °C between sonications). Proteinase K (PK) resistant prion protein was detected using immunoblotting with antibodies 8G8 and BAR224.

**References**

1. Smith CB, Booth CJ, Wadzinski TJ, Legname G, Chappell R, Johnson CJ, et al. Humic substances interfere with detection of pathogenic prion protein. Soil Biology and Biochemistry. 2014;68:309-16.

2. Giachin G, Narkiewicz J, Scaini D, Ngoc AT, Margon A, Sequi P, et al. Prion protein interaction with soil humic substances: Environmental implications. PLoS One. 2014;9(6):e100016.

3. Safar J, Wille H, Itri V, Groth D, Serban H, Torchia M, et al. Eight prion strains have PrPSc molecules with different confirmations. Nature nedicine. 1998;4(10):1157-65.

4. Johnson C, Johnson J, Vanderloo JP, Keane D, Aiken JM, McKenzie D. Prion protein polymorphisms in white-tailed deer influence susceptibility to chronic wasting disease. J Gen Virol. 2006 Jul;87(Pt 7):2109-14.

5. Lee IS, Long JR, Prusiner SB, Safar JG. Selective precipitation of prions by polyoxometalate complexes. J Am Chem Soc. 2005 Oct 12;127(40):13802-3.

6. Johnson CJ, Aiken JM, McKenzie D, Samuel MD, Pedersen JA. Highly efficient amplification of chronic wasting disease agent by protein misfolding cyclic amplification with beads (PMCAb). PLoS One. 2012;7(4):e35383.

7. Castilla J, Saa P, Hetz C, Soto C. In vitro generation of infectious scrapie prions. Cell. 2005 Apr 22;121(2):195-206.

8. Moudjou M, Sibille P, Fichet G, Reine F, Chapuis J, Herzog L, et al. Highly infectious prions generated by a single round of microplate-based protein misfolding cyclic amplification. MBio. 2014;5(1):e00829-13.

9. Cooke CM, Rodger J, Smith A, Fernie K, Shaw G, Somerville RA. Fate of prions in soil:  Detergent extraction of PrP from soils. Environmental Science & Technology. 2007 2007/02/01;41(3):811-7.
