# Supplementary figures and images for: Mineral licks as environmental reservoirs of chronic wasting disease prions
Source: PLoS One. 2018 May 2;13(5):e0196745. doi: 10.1371/journal.pone.0196745 (PMC5931637; doi:10.1371/journal.pone.0196745)

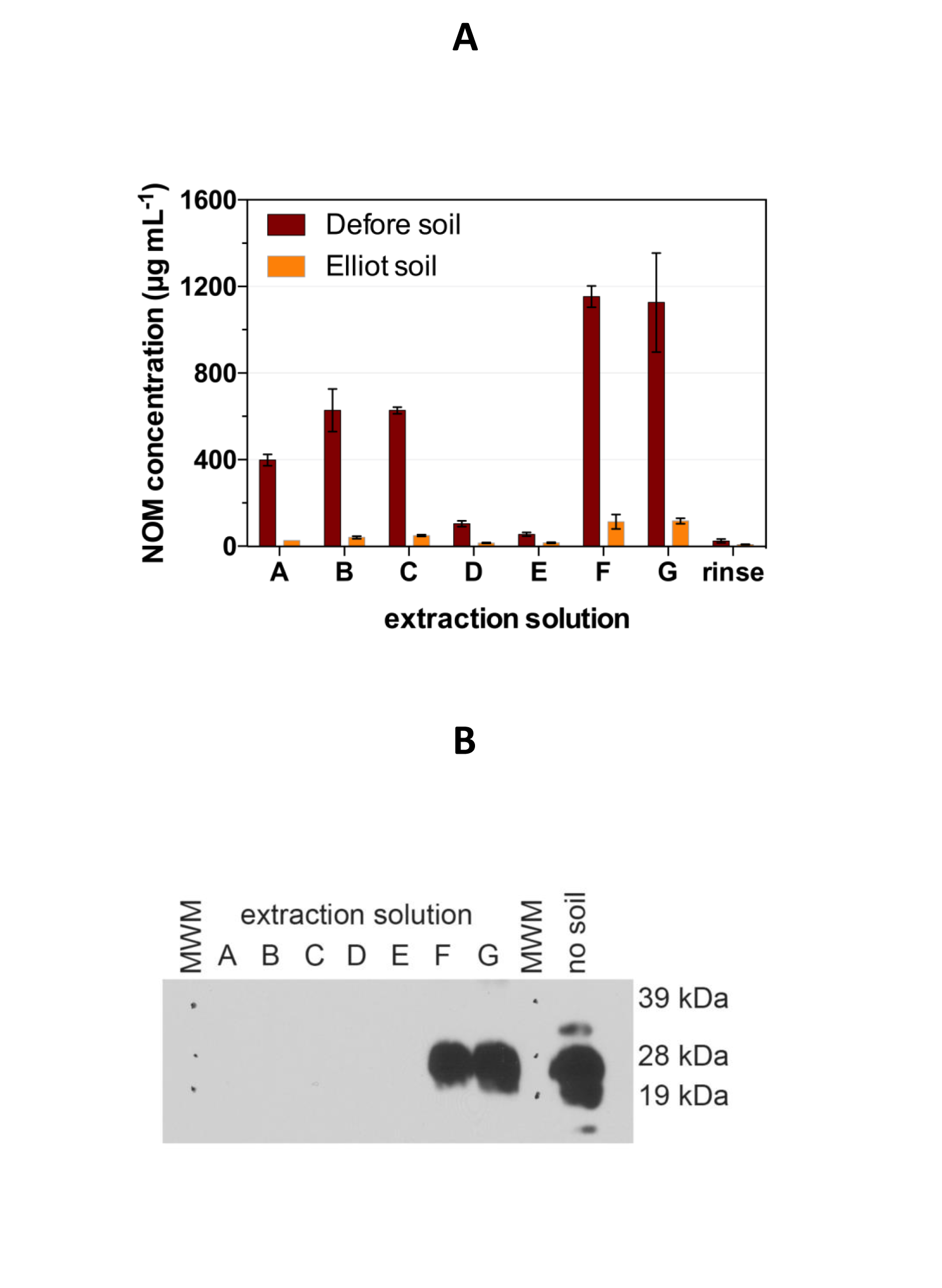

Supplement: S1 Fig — (A) Estimated amount of NOM extracted from Defore and Elliot soils using the indicated buffers. Samples of Defore or Elliot soil (25 mg) were rinsed with ultrapure water (200 μL each) with shaking (2 h, 1,200 RPM, room temperature). Samples were centrifuged (1000g, 10 min), and the supernatant was removed and saved as water rinse. The indicated extraction solution was added (200 μL) to each of the soil pellets and vortexed (2 h, 1,200 RPM, room temperature). Soil particles were sedimented (1000g, 10 min), and the supernatants were retained. Extractions were done in triplicate, and 100 μL of each extract and the water rinses were used to assay the absorbance at λ = 465. Absorbances were compared to dilution series of Elliot soil humic acid in each of the above extraction solutions, and the concentration of NOM was estimated. Shown are the mean NOM concentrations for the three replicates with the standard deviations. (B) Extraction of PrPCWD from soil. PK-treated 10% brain homogenate from a CWD-positive white-tailed deer (40 μL) was adsorbed to Elliot soil (25 mg) in ultrapure water (100 μL, 24 h) followed by a 2-h desorption step in 100 μL water (to remove any non-adsorbed unbound PrPCWD). The sorbed PrPCWD was extracted at room temperature with 200 μL of the indicated extraction solution and analyzed by immunoblotting. Abbreviations: A-G, extraction solutions (see descriptions above); M, molecular mass marker; mAb, monoclonal antibody; rinse, water rinse; S, supernatant from binding experiment. (TIF) [file pone.0196745.s002.tif]

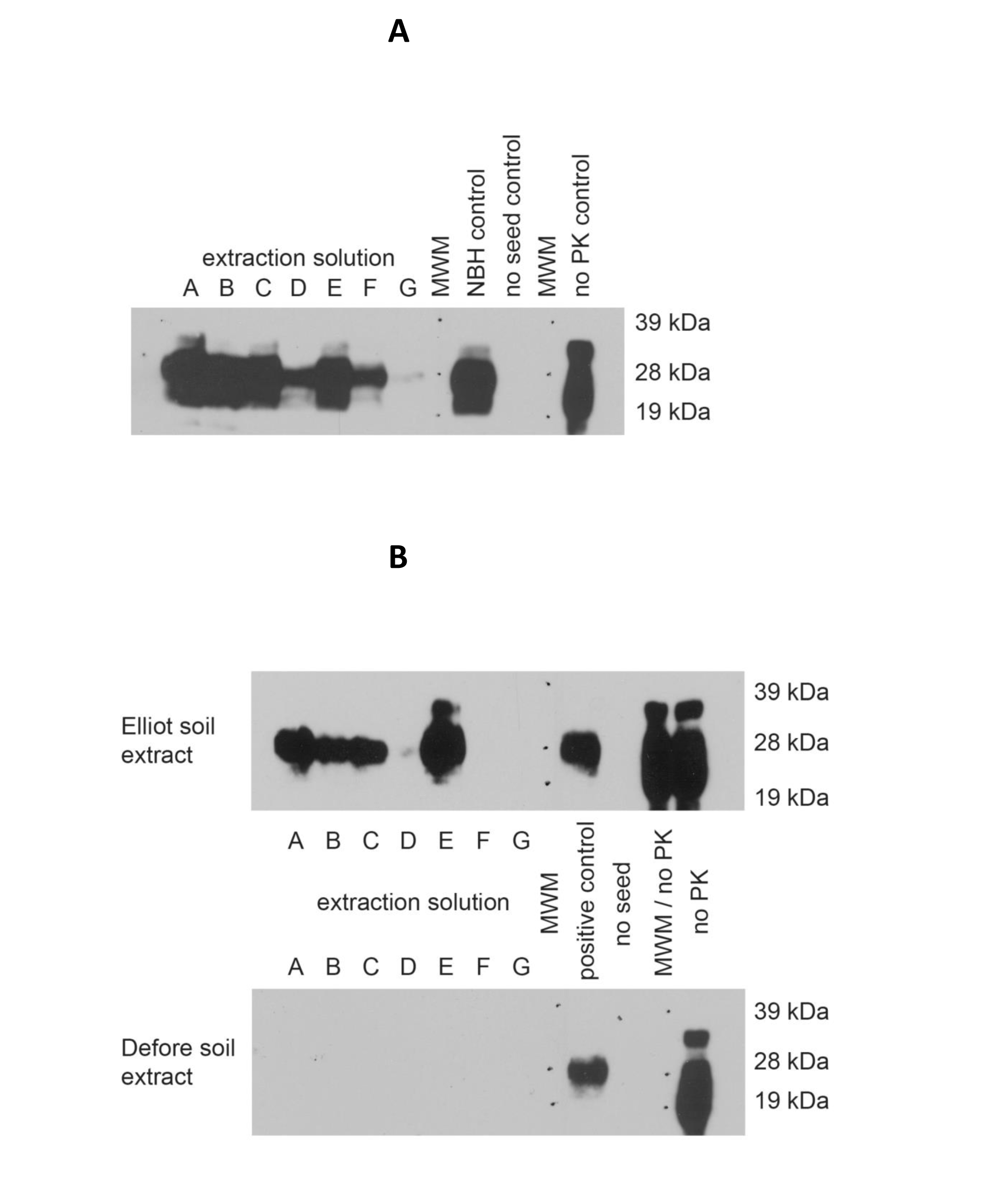

Supplement: S2 Fig — (A) Extraction solutions (8 μL) were mixed with 2 μL from the second 5-fold dilution of 10% brain homogenate (BH) from an end-staged CWD-positive wt/wt deer. Normal brain homogenate (NBH; 90 μL) was added with two Teflon® beads in a 200-μL thin-walled PCR tube. Samples were sonicated for 96 cycles (30 s sonication with 27:30 incubation at 37 °C between sonications). (B) Elliot soil or Defore soil (25 mg) was extracted using 100 μL of the indicated extraction solution. An aliquot (8 μL) of these extracts were mixed with 2 μL from the second 5-fold dilution of 10% BH from an end-stage CWD-positive wt/wt deer. NBH (90 μL) was added with two Teflon® beads in a 200-μL thin-walled PCR tube. Samples were sonicated for 96 cycles (30 s sonication with 27:30 incubation at 37 °C between sonications). Extraction solutions are described in the text. Proteinase K (PK) resistant prion protein was detected using Western blot with antibodies 8G8 and BAR224. (TIF) [file pone.0196745.s003.tif]

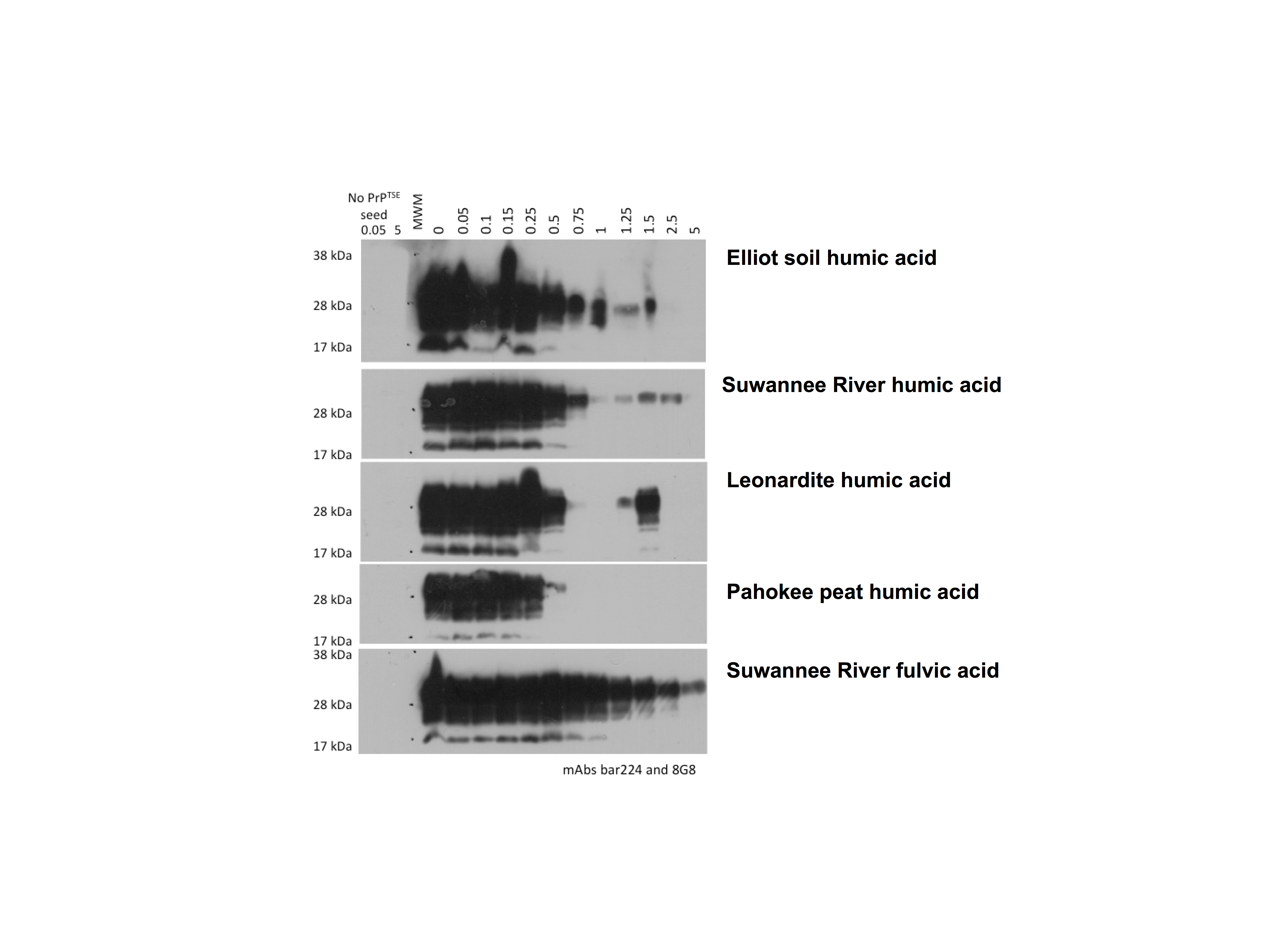

Supplement: S3 Fig — Prions (10% CWD brain homogenate) were diluted using four serial fivefold dilutions in normal brain homogenate (NBH) to obtain the seed dilution used in this experiment. The seed dilution (2 μL) was transferred into 36 μL NBH with saponin, and 2 μL humic or fulvic acid (dissolved in ultrapure water) was added to achieve a total mass of 5, 2.5, 1.5, 1.25, 1, 0.75, 0.5, 0.25, 0.15, 0.1, 0.05 or 0 μg of the indicated humic or fulvic acid. All samples were subjected to a single round of mb-PMCA for 96 cycles. Immunoblots shown are representative of three replicates and were probed with antibodies BAR224 and 8G8. (TIF) [file pone.0196745.s004.tif]

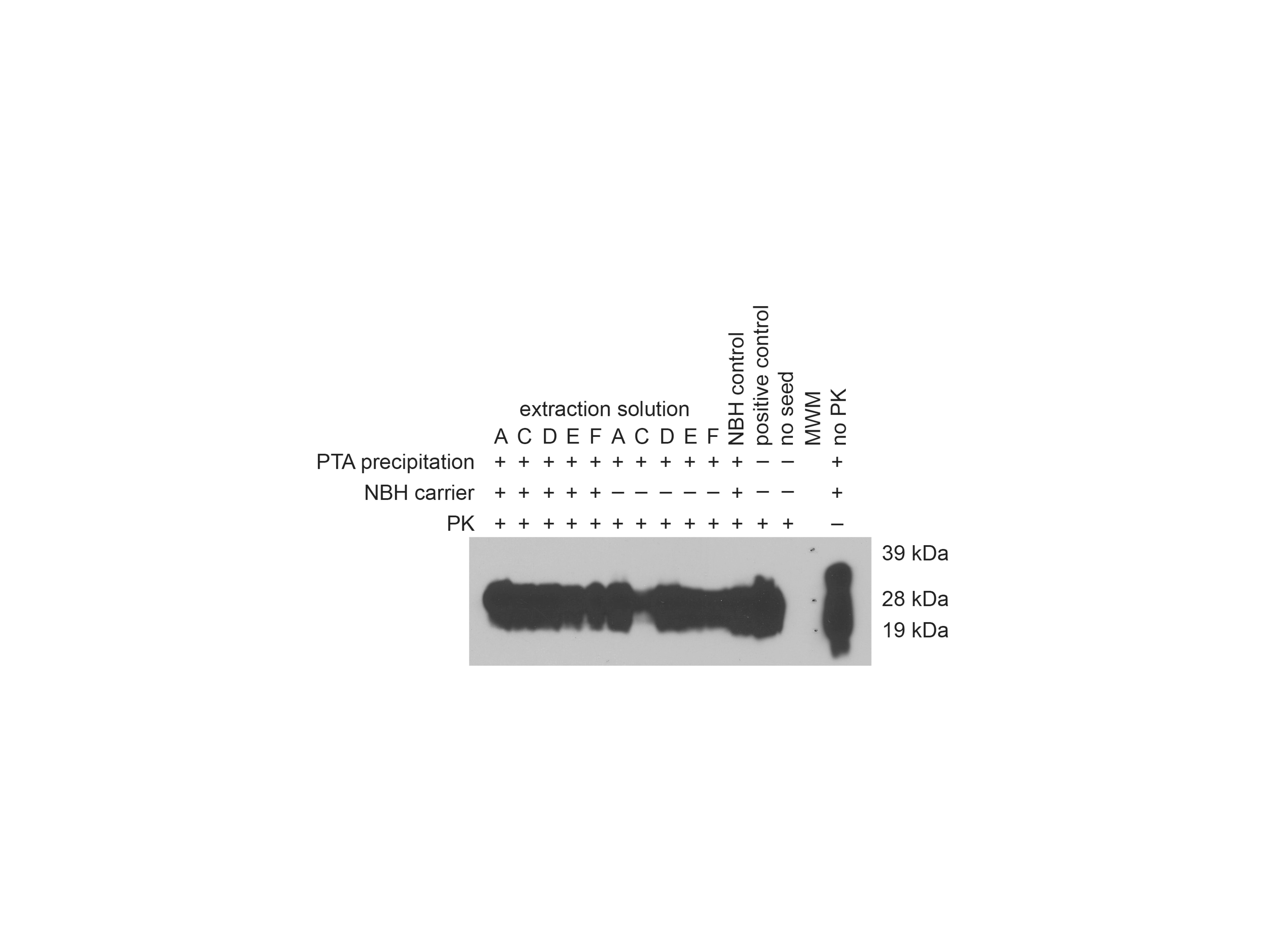

Supplement: S4 Fig — After NaPTA precipitation, 10 μL sample extracts from each extraction solution indicated were added to a PMCAb reaction. Extraction solutions were (A) 0.1 M sodium phosphate buffer, pH 7.4; (C) 1% NP40 in 0.1 M sodium phosphate buffer, pH 7.4; (D) McDougall’s buffer, pH 8.23; (E) PMCA buffer (1% Triton X-100 in PBS, pH 7.4 with 0.15 M NaCl and 0.05% saponin); and (F) 1% sarkosyl in 0.1 M sodium phosphate buffer, pH 7.4. Extraction solutions (B) and (G) not in immunoblot. Samples, normal brain homogenate (NBH; 90 μL), and two Teflon® beads were added to a 200-μL thin-walled PCR tubes containing NBH. Samples were sonicated for 96 cycles (0.5 min sonication with 27.5 min incubation at 37 °C between sonications). Proteinase K (PK) resistant prion protein was detected using immunoblotting with antibodies 8G8 and BAR224. (TIF) [file pone.0196745.s005.tif]
